# Laser–Metal Interaction with a Pulse Shorter than the Ion Period: Ablation Threshold, Electron Emission and Ion Explosion

**DOI:** 10.3390/nano13111796

**Published:** 2023-06-03

**Authors:** Eugene G. Gamaly, Saulius Juodkazis

**Affiliations:** 1 Laser Physics Centre, Department of Quantum Science and Technology, Research School of Physics, The Australian National University, Canberra, ACT 2601, Australia; 2 Optical Sciences Centre (OSC) and ARC Training Centre in Surface Engineering for Advanced Materials (SEAM), School of Science, Swinburne University of Technology, Hawthorn, VIC 3122, Australia; sjuodkazis@swin.edu.au; 3 WRH Program International Research Frontiers Initiative (IRFI), Tokyo Institute of Technology, Nagatsuta-cho, Midori-ku, Yokohama 226-8503, Kanagawa, Japan

**Keywords:** ultra-short laser pulses, laser pulses shorter than the ion period, non-equilibrium ablation, Coulomb explosion, micromachining, THz emission

## Abstract

The laser energy per unit surface, necessary to trigger material removal, decreases with the pulse shortening, becoming pulse–time independent in the sub-picosecond range. These pulses are shorter than the electron-to-ion energy transfer time and electronic heat conduction time, minimising the energy losses. Electrons receiving an energy larger than the threshold drag the ions off the surface in the mode of electrostatic ablation. We show that a pulse shorter than the ion period (Shorter-the-Limit (StL)) ejects conduction electrons with an energy larger than the work function (from a metal), leaving the bare ions immobile in a few atomic layers. Electron emission is followed by the bare ion’s explosion, ablation, and THz radiation from the expanding plasma. We compare this phenomenon to the classic photo effect and nanocluster Coulomb explosions, and show differences and consider possibilities for detecting new modes of ablation experimentally via emitted THz radiation. We also consider the applications of high-precision nano-machining with this low intensity irradiation.

## 1. Introduction

Coulomb forces are entirely responsible for keeping a solid intact. Quantum effects in solids significantly modify the Coulomb interactions. In the unperturbed metal, the wave functions of conduction electrons are periodic Bloch waves, allowing the conduction electrons to propagate through the metal without perturbation by attraction from the ion cores. Intense (∼$10^{14}$ W/cm^2^) sub-picosecond laser pulses excite electrons, while the ion’s cores remain unperturbed. The conduction electrons oscillate with energy comparable to the Fermi energy, violating the Braggs conditions, destroying the Bloch wave function, and converting the metal into plasma. The multi-particle Coulomb interaction between electrons and ions is restored over a certain duration depending on the electron-to-ion mass ratio. This time is in the range of a few femtoseconds to a few tens of femtoseconds depending on the ion’s mass.

A sub-picosecond pulse duration (from a few tens of picoseconds to a few tens of femtoseconds) is shorter than the electron-to-ion energy transfer time and electronic heat conduction time. It is established experimentally [1,2] that the energy density per unit area triggering material ejection (the threshold fluence) does not depend on the pulse duration. The analysis shows that the collective electrostatic field of hot electrons drags cold ions off the skin layer of a laser-excited solid [3]. The energy of an electron is proportional to the absorbed energy per unit surface. The threshold achieved when the absorbed energy per electron in the outmost surface layer is equal to the sum of the work function of the electron and the cohesion (binding) energy of the atom. The calculated threshold coincides well with the measured data [3]. Hence, it is obvious that the laser–matter interaction in this mode occurs over a longer time than necessary for the collective electrostatic field restoration.

**Figure 1:**
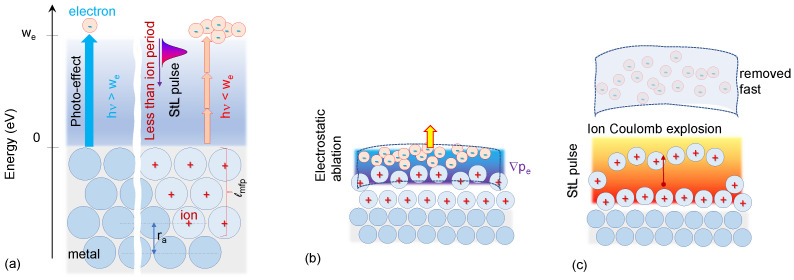
The schemes of the different interaction regimes. (**a**) **Left**: a knocked-off electron by a single UV photon in the classic photo effect; **Right**: absorption of multiple photons of an StL laser pulse followed by an electron ejection. (**b**) In electrostatic ablation, the ions are driven by the gradient of the electronic pressure, $\nabla p_{e}$. (**c**) In StL interaction mode, the ions are driven by the Coulomb repulsion layer by layer, with the outmost surface layer moving first and fastest. The energy scale is the work function, $w_{e}$; the space scales are the mean free path of an electron, $l_{mfp}$, and inter-atomic distance, $r_{a}$.

The subject of this paper is the analysis of the laser–metal interaction with a pulse duration shorter than the time necessary for the collective electrostatic field restoration. First, we identify the limit of the restoration time in a solid and in a plasma. Then, we analyse the electrostatic ablation mode and compare it to the shorter-the-limit (StL) pulse–metal interaction. The laser-excited conduction electrons in this mode have no time to affect the ion’s cores. Therefore, an electron with an energy larger than the work function leaves the surface with the kinetic energy $\epsilon_{kin}=\epsilon_{e}-w_{e}$, like in the classical photo effect ($\epsilon_{e}$ is the energy of electron and $w_{e}$ is its work function; Figure 1).

However, in Millikan’s experiments [4] (Nobel prize 1923), the intensity of the UV photons, $\hbar \omega >w_{e}$, i.e., the energy of photons per unit surface and unit time, was very low $10^{-7}$–$10^{-3}$ W/cm^2^. Hence, a photon interacted with an electron as a particle, swiftly knocking it off the potential well with a depth equal to the work function. The number density of affected ions was much lower than the initial density and the spatial separation of ions was very large, preventing any ion-to-ion interaction. The long interaction time (minutes) allowed the weak electric currents from the metal bulk to restore the neutrality.

Unlike the photo effect, during more intense StL–metal interactions, the electrons have an energy larger than the work function from the flow of photons as electro-magnetic waves at $\hbar \omega \ll w_{e}$, while the unperturbed positively charged ions remain immobile inside a few close-to-the-surface atomic layers during the interaction time, which is shorter than that necessary for restoring the collective Coulomb force of ions. After the end of the pulse, the joint electric field of the positive charges ejects the surface ions. The acceleration of ions, which is proportional to the surface charge density, exceeds that of electrostatic ablation by two orders of magnitude. Electron emission is followed by ion explosion, ablation, and THz radiation from the expanding plasma. We discuss the experiments for the possible verification of the StL pulse action and consider applications of high-precision nano/micro-machining.

## 2. Hierarchy of the Time Scales in the Electron–Ion Interactions

There are two domains of electron–ion Coulomb interactions. The first domain includes particle-to-particle collisions of momentum and energy exchange in plasma. The second group deals with the collective electron–ion interactions in plasma and solids when a particle is under the action of multiple fields of surrounding positive/negative charges.

Time scales in plasma are straightforward. The shortest is the electron–electron (e-e) and electron–ion (e-i) momentum exchange time, $t_{m}=\nu_{ei}^{-1}\propto \frac{\epsilon_{e}^{3/2}}{n_{e}}>\omega_{pe}^{-1}$, where $\nu_{ei}$ is the frequency of e-i collisions, $n_{e}$ is the number density of electrons, and $\omega_{pe}$ is the electron plasma frequency. The maximum momentum exchange rate is around the electron plasma frequency. Hence, the momentum exchange time is around ∼0.1 fs for the solid density plasma. The energy exchange time is larger by the ratio of ion mass $M_{ion}$ to the electron mass $m_{e}$, namely $t_{m}^{en}=\nu_{ei}^{-1}\frac{M_{ion}}{m_{e}}$. It is in the range of a few ps for the mass ratio of $10^{5}$. We start with the collective interactions in plasma where the physics is straightforward.

### 2.1. Time Scales for the Electron–Ion Collective Coupling in Plasma

In cold, non-magnetic quasi-neutral plasma with an equal number of positive and negative charges, an unbalanced force always acts on each charge from the neighbours, causing the charge to move and therefore the electrons density to fluctuate (a direct consequence of the Gauss theorem). The sum of electro-static fields forces the electrons to oscillate with the electron plasma frequency $\omega_{pe}^{2}=\left(4\pi e^{2}n_{e}\right)/m_{e}$ after the time $t_{C,e}\approx \omega_{pe}^{-1}$; in SI units $\omega_{pe}^{2}=e^{2}n_{e}/\left(\varepsilon_{0}m_{e}\right)$ with $\varepsilon_{0}\equiv \frac{1}{\mu_{0}c^{2}}$ (the conversion between proportionality constants of the Coulomb’s force acting on separated electric charges and magnetic force between currents is $2k_{el}=c^{2}k_{mag}$, where the electric and magnetic constants $k_{el,mag}$ depend on the definitions (units) of charge and current, respectively, $k_{el}=1$ (CGS) and $k_{el}=\frac{\mu_{0}c^{2}}{4\pi }$ (SI)). Thus, the electrons start oscillating after the time $t_{C,e}$, from the moment the electronic plasma is instantaneously created ($∼10^{-16}$ s). The sum of the electro-static fields of the oscillating electrons forces heavy ions to oscillate with the ion plasma frequency $\omega_{pi}^{2}=\left(4\pi e^{2}n_{i}\right)/M_{i}$. The time when the Coulomb fields of the multiple electrons start affecting the ions is $t_{C,i}\approx \omega_{pi}^{-1}=t_{C,e}\sqrt{M_{i}/m_{e}}∼10^{-14}$ s. The electron plasma frequency from Al to Ag is in the range $\left(1.97-1.21\right)\times 10^{16}$ s^−1^, defining the time when motion of ions begins as $t_{C,i}\approx \left(0.5-0.82\right)\times 10^{-16}\sqrt{M_{i}/m_{e}}$ (s).

### 2.2. Time for the Perturbation of Ion Core Positions in a Cold Solid

At zero approximation the ions are assumed immobile and electrons are moving in the Coulomb field of the stationary ions. The full energy of a solid (a non-relativistic Hamiltonian in the quantum treatment) includes the kinetic energy of electrons and ions, electron–electron and ion–ion Coulomb interactions, and electron–ion interactions [5]. The effective potential energy as well as the energy of the Coulomb interaction between the nuclei in equilibrium is approximated well by the energy of interaction of two charges, $U_{0}\approx \frac{e^{2}}{2r_{B}}=\frac{m_{e}e^{4}}{2\hbar^{2}}$, where $r_{B}$ is the Bohr radius. The ion core motion under the action of the electron’s Coulomb field in a cold solid can be considered as a perturbation. It is the second term in the expansion of the core’s potential energy in the series on the deviation of the ion core’s position from the equilibrium, δR. The expansion of the potential near the equilibrium reads:

(1) $$U=U_{0}+\delta U_{0}\approx U_{0}+\frac{1}{2}\frac{\partial^{2}U\left(R\right)}{\partial R^{2}}\delta R^{2}.$$

The second term is the potential energy of nucleus oscillations:

(2) $$\delta U_{0}\approx \frac{U_{0}\delta R^{2}}{2r_{B}^{2}}.$$

The minimum momentum of a nucleus is estimated from the uncertainty relations, $p_{i}\geq \hbar /\delta R$. Then, the ion’s kinetic energy follows: $p_{i}^{2}/2M_{i}\geq \hbar^{2}/\left(2M_{i}\delta R^{2}\right)$. Equalising the potential energy to the kinetic energy of the core (the Virial theorem), one obtains the nucleus displacement, $\delta R\approx {\left(2m/M_{i}\right)}^{1/4}\times r_{B}$. Hence, the ion’s energy increase from Equation (2) is:

(3) $$\delta U_{0}=\epsilon_{ion}\approx \frac{U_{0}}{2}\sqrt{\frac{2m_{e}}{M_{i}}}.$$

The minimum time when ion starts moving after receiving the energy from the electron’s Coulomb field follows from the uncertainty relation:

(4) $$t_{ion}\approx \frac{\hbar }{\epsilon_{ion}}=\frac{\sqrt{2}\hbar }{U_{0}}\sqrt{\frac{M_{i}}{m_{e}}}=\sqrt{2}t_{at}\sqrt{\frac{M_{i}}{m_{e}}},$$

where the characteristic atomic time is $t_{at}=\hbar /U_{0}=0.48\times 10^{-16}$ (s) for $U_{0}=13.6$ eV. Thus, $t_{ion}\approx \sqrt{\frac{M_{i}}{m_{e}}}\times 0.68\times 10^{-16}$ (s). This estimate is based only on the fundamental constants and mass ratio. Therefore, it sets the minimum time for an electron to affect an ion by its electric field in the solid.

One can see the proximity of this result to the estimate for the solid density plasma, $t_{C,i}\approx \left(0.5-0.82\right)\times 10^{-16}\sqrt{\frac{M_{i}}{m_{e}}}$ (s). In plasma, this time depends on the electron’s number density, explicitly reflecting the collective nature of the effect that is implicit in the above estimate. It is legitimate to assume that in a shorter time than that required for the sum of forces by chaotically directed fields of multiple charges to build up, the ions are not moving. Hence, the electrons excited during the period shorter than $t_{C,i}$ do not interact with the core ions.

## 3. Ablation of a Metal by Different Pulses: Long (Electrostatic Mode) and Short (StL Mode)

Let us compare the ablation of a metal in two different experiments by two pulses (both shorter than the electron-to-ion energy transfer time) of different durations delivering the same energy per pulse at the same focal spot (the same fluence). The longer pulse interacts in the regime of electrostatic ablation, while the shorter pulse ablates the same metal in the StL interaction mode. The electron temperature distribution in the skin layer depends on the absorbed fluence. The number of conduction electrons remains practically unchanged in the considered intensity range. Therefore, the assumption that the absorption coefficient and skin length at two different intensities are approximately the same is reasonable. The processes in the considered experiments depend only on the ablation mode (pulse duration). The pulses are of high contrast (no pre-plasma, step-like density gradient to the end of the pulse) incident along the normal to the surface. The laser energy is absorbed by the conduction electrons in the skin layer. The solution of the 1D Maxwell equation in a metal allows calculation of the absorbed energy density through the Poynting vector. Then, from the energy equation for electrons follows the electron energy space and time dependence in the form:

(5) $$\epsilon_{e}\left(x,t\right)=\epsilon_{e}\left(0,t\right)\times e^{-\frac{2x}{l_{s}}},$$

where $\epsilon_{e}\left(0,t\right)=\frac{2A}{n_{e}l_{s}}F\left(0,t\right)$ and the fluence is a time integral of intensity $F\left(0,t\right)=\int_{0}^{t}I\left(0,\tau \right)d\tau$. Here, *A* is the Fresnel absorption coefficient, the ratio of the absorbed to the incident energy, $l_{s}$ is the skin length for E-field, and $n_{e}$ is the electron number density (see details in Appendix A, Appendix B, Appendix C, Appendix D, Appendix E, Appendix F, Appendix G and Appendix H).

### 3.1. Electrostatic Ablation

The pulse duration for the electrostatic ablation should be in the range $t_{C,i}<t_{pulse}<t_{m}^{en}$, larger than that for building the collective Coulomb force and much less than the electron-to-ion energy transfer time. Electrostatic ablation of a metal surface has been experimentally verified by 15 fs laser pulses [6]. The threshold fluence from Equation (5) is defined as the electron energy necessary for removal of an ion from the outermost surface layer. This energy is equal to the sum of the cohesion (binding) energy and work function, $\epsilon_{e}\left(0,t_{p}\right)=\epsilon_{b}+w_{e}$. The threshold for metals, $F_{th}\left(0,t_{p}\right)=\frac{\left(\epsilon_{b}+w_{e}\right)n_{e}l_{s}}{2A}$, agrees well with the measurements [1,2]. Let us define the ablation depth $l_{abl}$ from the similar condition, $\epsilon_{e}\left(l_{abl},t_{p}\right)=\epsilon_{b}+w_{e}$. Then, the energy of an electron in the outermost surface layer is $\epsilon_{e}\left(0,t_{p}\right)=\left(\epsilon_{b}+w_{e}\right)e^{2}$, and the incident laser fluence is $F=e^{2}F_{th}\left(0,t_{p}\right)$, where e=2.71 is Napier’s number.

The momentum equations for electrons and ions are the following:

(6) $$\begin{array}{ccc} m_{e}\frac{\partial v_{e}}{\partial t} & = & eE_{elst}+\frac{1}{n_{e}}\nabla p_{e}, \end{array}$$

(7) $$\begin{array}{ccc} M_{i}\frac{\partial v_{i}}{\partial t} & = & eE_{elst}, \end{array}$$

where $p_{e}$ is the electron pressure and $E_{elst}$ is the electroscatic field driving ablation (Figure 1b). Here, the electric field is the coarse-grained field associated with the collective interaction of plasma charges [7]. In considering the ion’s motion, the electron’s inertia can be ignored, $n_{e}eE_{elst}\approx -\nabla p_{e}$. The collective action of hot electrons drives the cold ion’s motion off the metal:

(8) $$M_{i}\frac{\partial v_{i}}{\partial t}=eE_{elst}=-\nabla \epsilon_{e}=\frac{2\epsilon_{e}}{l_{s}}.$$

Electronic heat conduction smooths the gradient well after the end of the pulse. The cooling time, when the gradient along with the ions acceleration goes to zero, is $t_{cool}=l_{s}^{2}/D_{heat}$, where the diffusion coefficient is defined by the e-i momentum exchange rate $\nu_{ei}^{mom}$ as $D_{heat}=v_{e}^{2}/\left[3\nu_{ei}^{mom}\right]$. It is in the picoseconds range. Thus, the number of ablated ions (focal area is known), their acceleration, and their final velocity can be calculated.

### 3.2. Ultra-Short Pulse $t_{C,i}>t_{pulse}$ Interaction

Let us now consider a much shorter pulse, $t_{C,i}>t_{pulse}$, interaction with the same metal and the same absorbed surface energy density. The energy and space distribution of electrons created by the long and short pulse is the same. Now, the conduction electrons can get an energy larger than the work function during the period shorter than that necessary for building the link to ions. The state is very similar to that in the classical photo effect, with the difference that this state has been created by multi-photon absorption (Figure 1). The electrons in a few atomic layers at the distance of the electronic mean free path from the metal–vacuum boundary can escape the metal with the kinetic energy $\epsilon_{kin}=\epsilon_{e}-w_{e}$, leaving *N* atomic layers with positively charged ions. This number is equal to the electron’s mean free path divided by the atomic monolayer thickness, $N=l_{mfp}/r_{a}$. The focal spot area, $S_{f}$, with the depth $l_{mfp}$, and atomic number density $n_{a}$, became positively charged with the surface charge density, $\sigma =en_{a}N\times r_{a}$ (Figure 1). In accordance with electrostatics [8], the charged thin infinite plate creates equal electric fields perpendicular to the plane in the positive and negative directions. Each field is proportional to the surface charge density, $E_{els}=2\pi \sigma$. Thus, a single charged ion of mass $M_{i}$ is under action of this force pulling ion off:

(9) $$\frac{dv_{ion}}{dt}=2\pi e^{2}n_{a}N\times \frac{r_{a}}{M_{i}}=N\omega_{pi}^{2}r_{a}/2.$$

Note that the maximum acceleration of two repelling ions (at small displacements, $\zeta \ll r_{a}$) is equal to $\omega_{pi}^{2}r_{a}/3$ [9], while in the ideal case of an electrostatic charged plate, the acceleration is constant in space. The acceleration by a charged plate is enhanced by the multiple contributions of the surface charges. However, the acceleration is not constant in space in reality. The force has a maximum in the central part of the focal spot, decreasing with the distance to the focal boundary.

For the finite plate in plasma, the space scale, where it is reasonable to consider this acceleration as constant, must be much less than the size of the plate (the focal spot). Taking conservatively this scale as $r_{a}$, one gets the maximum velocity as $v_{i,max}\approx \sqrt{N\omega_{pi}^{2}r_{a}^{2}}$. For Ag ($\omega_{pi}=2.53\times 10^{13}$ s^−1^, $r_{a}=1.59\times 10^{-8}$ cm, N∼9), the velocity range is $v_{i,max}=\left(12-4\right)\times 10^{5}$ cm/s and the acceleration range is $\left(5-0.45\right)\times 10^{19}$ cm/s^2^, orders of magnitude larger than in electrostatic ablation (Figure 1).

Hence, there are a few major differences with electrostatic ablation. First, some of the electrons are ejected most probably isotropically. Second, some of the bare ions are accelerated by the Coulomb repulsion with an acceleration a couple of orders of magnitude higher than that during electrostatic ablation. Third, the ablation threshold was reached at an electron energy slightly above the work function and caused electrons to leave from a couple of the outermost surface layers, which then triggered ions to be repulsed.

## 4. Radiation from the Ablated Plasma

The ablated plasma is a current flowing mainly perpendicular to the sample surface, decelerating slowly before attaining constant velocity. Let us compare the radiation of plasma flow in two ablation modes. We consider ablation of an Ag target with the same incident and absorbed surface energy density producing the identical space energy distribution and ablation depth equal to the skin depth. The total number of ablating particles in both cases is the same. However, in the case of an StL pulse there are two groups of ions with different accelerations.

### 4.1. Radiation from the Electrostatically Ablated Plasma

The frequency spectrum and total power of radiation emitted by the time-dependent current depends on the full number of emitting charges, their acceleration, and its duration. Ag ions’ acceleration in the electrostatic ablation mode is $\frac{\partial v_{i}}{\partial t}=\frac{2\epsilon_{e}}{M_{i}l_{s}}=2.3\times 10^{17}$ cm/s^2^, while the number of ablated ions is $N_{abl}=2.5\times 10^{11}$ ions (see Appendix A, Appendix B, Appendix C, Appendix D, Appendix E, Appendix F, Appendix G and Appendix H for details).

The Fourier transform of the time-dependent current defines the frequency spectrum of the emitted radiation: $j_{\omega }=\int j\left(t\right)e^{i\omega t}dt$. The acceleration of ions decreases because the electronic heat conduction leads to the flattening of the electronic pressure gradient. The acceleration decreases from the maximum to zero due to the skin layer cooling; the characteristic time, T∼1 ps, and inverse are a characteristic frequency of emitted radiation of ∼1 THz. The direction of the emission is perpendicular to the direction of non-relativistic acceleration. The power of the radiation emitted by the current is given by the Larmor formula [10]:

(10) $$P=\frac{2}{3c^{3}}{\left(\ddot{d}\right)}^{2},$$

where $\ddot{d}=\sum e\dot{v}$ is the sum taken over all dipoles’ acceleration, $\dot{v}$, in the current. The power of the emitted radiation is proportional to the square of the particles number and their deceleration. One can estimate the total energy emitted by the current using the following:

(11) $$\epsilon_{Rad}=T\times P\approx \frac{2T}{3c^{3}}{\left(eN_{abl}\frac{dv_{i}}{dt}\right)}^{2}.$$

Taking the acceleration as $2.3\times 10^{17}$ cm/s^2^, $N_{abl}=2.5\times 10^{11}$ ions, and $T∼10^{-12}$ s, one gets total energy of radiation as $1.88\times 10^{-12}$ J (a power of 1.88 W). Hence, the ratio of the total emitted energy to laser energy of 3 μJ is $∼6\times 10^{-7}$.

### 4.2. Radiation from the Plasma Ablated by StL Pulse

The total number of ablated atoms remains the same. However, the structure of the plasma outflow is different. First, the electrons from a few atomic layers with the thickness of the electronic mean free path are moving out. Then, the bare ions abandoned by electrons are expelled by repulsion. Finally, the bulk of the ablated material is removed with low velocity and the features considered in the previous paragraph.

The number of ions in layers from where electrons escaped is $N_{exp}=\left(l_{mfp}/l_{s}\right)N_{abl}∼7.5\times 10^{9}$. The mean free path of an electron $l_{mfp}=\nu_{e}/\nu_{ei}^{mom}∼1.48\times 10^{-7}$ cm extends to nine monolayers. The electrons from these layers are emitted during the StL pulse. The removal of ions by repulsion occurs layer by layer with gradually decreasing acceleration in accordance with the diminishing number of layers:

(12) $$\frac{dv_{ion}}{dt}=N\omega_{pi}^{2}r_{a}/2.$$

For Ag, the acceleration range of exploding ions is $10^{19}$–$10^{20}$ cm/s^2^, while the velocity range is $v_{i,max}=\left(12-4\right)\times 10^{5}$ cm/s. The time for reaching the constant velocity of expansion is a few hundred fs (100–400 fs). Therefore, the frequency of radiation is in the range of (10–2.5) THz. Now, estimates for the power and total energy of radiation are straightforward by Equations (10) and (11): P=(3.2−320) W and the total energy (taking $T=2.5\times 10^{-13}$ s) is around $2.5\times 10^{-11}$ J. Hence, only 3% of ablated ions emit by the order of magnitude larger energy in a slightly higher frequency range. The plasma plume produced by the StL pulse contains a major part of slow ions, emitting less than 10% radiation in the THz range. About ∼3% of the total ablated fast ions emit major radiation in a slightly higher frequency range. Two radiation peaks of different height are separated in time.

## 5. Discussion and Conclusions

The non-relativistic StL laser pulse (ponderomotive potential 10–20 eV) excites free electrons in the conduction band of metals during a time shorter than that required for the restoration of electron–ion Coulomb coupling. Excited electrons escape from a few close-to-the-surface atomic layers after gaining energy larger than the work function. These layers become positively charged and create an electrostatic field perpendicular to the surface, accelerating and ejecting ions by the Coulomb explosion. Let us discuss the similarities and differences of this phenomenon in regard to the classical photo effect and the Coulomb explosion of nanoclusters.

In the classic photo effect, a UV photon with an energy larger the work function knocks the conduction electron off the surface in a single photon–electron collision (Figure 1). To consider a photon as a quantum particle, the number of photons per cube of photon wavelength should be less than one [11]. The intensity in Millikan’s experiments [4] was in the range of $10^{-7}-10^{-3}$ W/cm^2^, well in accordance with the above criterion, leaving the rest of the conduction electrons in a free state (not affected by the ion cores). Therefore, an electron after collision escapes the metal with a kinetic energy in accordance with the Einstein formula: $\epsilon_{kin}=\hbar \omega -w_{e}$.

In the StL pulse–metal interaction, the high-frequency laser field convert electrons into a plasma state, thus destroying the free electron–ion core relations of the metal, in contrast to the classical photo effect. However, an electron absorbs energy during a period shorter than that needed for the restoration of the collective electron–ion coupling. The electron which received energy more than the work function escapes the metal without being affected by the ion cores due to the extremely rapid nature of interaction. Ions in a few atomic layers are left immobile and charged. Then, the Coulomb repulsion drives ions out of the metal.

The phenomenon of the Coulomb explosion of small [12] and large molecules [13] and nanoclusters [14,15,16] has been studied for more than three decades. The relativistic laser beam with the ponderomotive potential in the order of MeVs ionises nanoclusters up to several electrons per atom. Electrons are accelerated and swiftly ejected from a cluster with an energy comparable to the ponderomotive potential, leaving ions immobile and positively charged. The cluster acquires a big total positive charge, resulting in the Coulomb explosion. The kinetic energy of exploded ions is on the order of MeVs depending on the total charge of the cluster. Recent studies provide new insights into ablation at ultra-relativistic intensities, showing a step-like electrostatic potential which drives the disassembly of the solid [17]. In the StL–metal interaction, similar events develop on a much smaller energy scale and on a similar short time scale. The laser-ejected electrons are followed by fast ions (a few percent) accelerated by the explosion and followed by the slow ions of conventional ablation. An analysis shows that the ablated flow of fast ions is a more intense source of THz radiation than the slow current. Using light metals (such as Al) and energetic lasers, one can create an StL-pulse-generated point source of THz radiation (controlled by the choice of metal, pulse duration, laser energy, and focal spot size).

Summing up the presented analysis suggests that an intense laser pulse with a duration less than the ion period (a few tens of fs) is capable of swiftly ejecting the conduction electrons from a few near-surface atomic layers, followed by the flow of energetic ions ejected by the Coulomb repulsion and emission of THz radiation. As it follows from the above, in this interaction regime (StL), it is possible to remove a few atomic layers from a metal by the action of a single laser pulse (see Appendix H). The number of atomic layers removed by explosion, i.e., the electron’s mean free path, is controlled by the absorbed surface energy density delivered by the laser. Indeed, the mean free path of electrons in the Coulomb collisions is $l_{mfp}\propto \epsilon_{e}^{2}$, while $\epsilon_{e}\propto F$. For experimental realisation and monitoring of the StL pulse ablation, detection of THz emission reported for the generation of single-cycle circularly polarised pulses under 40 fs pulsed irradiation of a water micro-flow [18] can be used.

## Data Availability

All data are provided within the main text.

## Appendix A. Classical Photo Effect

In Millikan’s experiments (1916), the photo effect was induced by UV radiation of the mercury discharge. The intensity at 365 nm (3.4 eV) was 10–$10^{4}$ erg/cm^2^s ($10^{-7}$–$10^{-3}$ W/cm^2^). The maximum flow of photons was $1.8\times 10^{15}$ photons/cm^2^/s. A UV photon swiftly knocks an electron out of the surface layer. Positive charges are equalised by the slow currents from the bulk. The quantum description of the photon as a particle is necessary when the number density of photons is less one photon per cubic of the photon’s wavelength [11].

## Appendix B. Properties of Metals at Room Temperature

The conduction electrons’ density $n_{e}$ is in $10^{23}$ cm^−3^, the inter atomic distance, $r_{a}={\left(4\pi n_{e}/3\right)}^{-1/3}$, is in $10^{-8}$ cm (Å), the plasma frequency is in $10^{16}$ s^−1^, and the effective electron’s mass is in units of free electron mass. The electron/ion mass ratio and ion frequency for Ag is $m_{e}/M_{i}=5.08^{-6}$ and $\omega_{pi}=2.53\times 10^{13}$ s^−1^ ($t_{Ci}=36.7$ fs).

**Table A1:** Parameters of metals which can be used for experiments with StL pulses. The maximum value of $w_{e}$ is shown and for which crystallographic plane [19].

Metal:	$n_{e}$	$M_{i}$	$r_{a}$	$\omega_{\mathrm{pe}}$	$m_{\mathrm{ef}}$	$C_{e}$	$\epsilon_{F}$	$\epsilon_{b}$	$w_{e}$
Units	$10^{23}$ cm^−3^	at.mass	Å	$10^{16}$ s^−1^	$m_{e}$	$\frac{3}{2}k_{B}$	eV	eV	eV; (max)
Al	1.806	26.982	1.09	1.97	1.48	0.46	11.63	3.065	4.2; (100)-plane
Cu	0.845	63.546	1.41	1.395	1.38	0.70	7.0	3.173	4.48–4.98
Au	0.59	196.97	1.38	1.126	1.14	0.69	5.52	3.81	5.47; (100)-plane
Ag	0.585	107.86	1.59	1.121	1.0	0.79	5.48	2.95	4.52–4.74

The electron heat capacity is estimated from $C_{e}\approx \frac{3}{2}k_{B}T_{e}\left(2x-x^{2}\right)$, where $x=k_{B}T_{e}/\epsilon_{F}$ attains the ideal gas value at x=1 [20] and $\epsilon_{F}$ is the Fermi energy. The electron heat capacity near the ablation threshold is $k_{B}T_{e}=\epsilon_{b}$, where $\epsilon_{b}$ is the binding energy, defined as the potential barrier against the free motion of atoms through the solid. For example, for Au, $\epsilon_{b}=3.81$ eV/atom (the cohesive energy per atom), $\epsilon_{F}=5.52$ eV, $x\approx \epsilon_{b}/\epsilon_{F}=0.69$, and $C_{e}=1.355$ ($k_{B}$), close to 1.5 as for the ideal gas.

## Appendix C. Optical Properties of the Electronic Plasma

The Drude-like dielectric permittivity reads [20]:

(A1) $$\varepsilon_{re}=1-\frac{\omega_{pe}^{2}}{\omega^{2}+\nu_{ei}^{2}},\;\;\;\;\varepsilon_{im}=\frac{\omega_{pe}^{2}}{\omega^{2}+\nu_{ei}^{2}}\times \frac{\nu_{ei}}{\omega },$$

and is related to the real and imaginary parts of the complex refractive index $\tilde{n}\equiv n+i\kappa$ via $\varepsilon_{re}=n^{2}-\kappa^{2}$ and $\varepsilon_{im}=2n\kappa$; $\kappa^{2}=\left(\right|\varepsilon_{re}\left|-\varepsilon_{re}\right)/2$ and $n^{2}=\left(\right|\varepsilon_{re}\left|+\varepsilon_{re}\right)/2$, where the complex modulus $\left|\varepsilon_{re}\right|=\sqrt{\varepsilon_{re}^{2}+\varepsilon_{im}^{2}}$. The Fresnel absorption coefficient reads $A=\frac{4n}{{\left(n+1\right)}^{2}+\kappa^{2}}$.

**Electron–ion momentum and energy transfer rates.** Near the maximum, it is defined as $\nu_{ei}\approx v_{e}/r_{a}$. Taking the electron velocity on the atomic scale, $2\times 10^{8}$ cm/s, one gets the rate of the order of the electron plasma frequency. We consider the conditions when the electron energy is of the same order of magnitude as the Coulomb energy and Fermi energy. The plasma is non-ideal and unscreened and the collision rate decreases. Cauble and Rozmus predicted [21] that in a solid density non-ideal plasma the e-i collision rate can be estimated as the following:

(A2) $$\nu_{ei}^{mom}\approx \omega_{ei}\frac{\ln\Lambda }{10N_{D}},$$

where the logarithm lnΛ and the number of particles in the Debye sphere $N_{D}$ for the ideal plasma are [7]:

(A3) $$\Lambda \approx \left(9N_{D}\right)/Z,\;\;\;N_{D}=1.7\times 10^{9}\sqrt{\theta_{e}^{3}/n_{e}},$$

where $\theta_{e}$ is the electron temperature and *Z* is the charge state. Taking $\epsilon_{e}=10$ eV and $n_{e}=0.585\times 10^{23}$ cm^−3^ (Ag), one obtains Λ=2 (ref. [10] gives 0.69), $N_{D}=0.22$, and $\nu_{ei}^{mom}\approx 0.3\omega_{ei}∼3\times 10^{15}$ s^−1^.

The electron-to-ions energy transfer time is $t_{ei}^{en}={\left[\nu_{ei}^{mom}\times \frac{m_{e}}{M_{i}}\right]}^{-1}$; for Ag, it is 65 ps.

At $\omega =1.88\times 10^{15}$ s^−1^ (wavelength of 1002 nm), $\omega_{pe}=1.21\times 10^{16}$ s^−1^, and $\nu_{ei}^{mom}\approx 3\times 10^{15}$ s^−1^, one gets $\varepsilon_{im}=16$ and $\varepsilon_{re}=-9$, i.e., n=2.16, κ=3.7, and A=0.365. The skin depth $l_{s}=43$ nm and the constant $C_{0}=\frac{2A}{n_{e}l_{s}}=3\times 10^{-18}$ cm^2^ is used in the calculation of electron energy (see below).

**Oscillation energy (ponderomotive potential)** is given by [22]: $\varepsilon_{osc}=9.375\left(1+\alpha^{2}\right)\times \frac{I}{10^{14}\;\left[W/{\mathrm{cm}}^{2}\right]}\times \lambda_{\mu m}^{2}$ (eV); here α=±1 for circular polarisation and α=0 for linear polarisation. The intensity *I* is averaged over many laser pulse periods.

## Appendix D. The Electron’s Energy Space and Time Dependence in the Skin Layer

The electron’s energy time/space dependence in the skin layer (normal skin) in 1D approximation follows from the energy equation under the assumption that the electron’s density and optical properties are time/space independent, and the pulse duration is much shorter the electron-to-ions energy transfer time, $t_{ei}^{en}={\left[\nu_{ei}^{mom}\times \frac{m_{e}}{M_{i}}\right]}^{-1}$. The electrons contain all the absorbed energy. The energy equation for electrons (no losses) is:

(A4) $$n_{e}\frac{\partial \epsilon_{e}}{\partial t}=Q_{abs}\left(x,t\right).$$

Here the absorbed energy density reads:

(A5) $$Q_{abs}\left(x,t\right)=\frac{2AI(t,0)}{l_{s}}e^{-2x/l_{s}}\;\;\left[W/{\mathrm{cm}}^{3}\right].$$

The Fresnel absorption coefficient *A* and skin depth $l_{s}$ are the following:

(A6) $$\frac{2A}{l_{s}}=\frac{4\omega \varepsilon_{im}}{c\times |1+\sqrt{\varepsilon }{|}^{2}},\;\;\;l_{s}=\frac{c}{\omega \kappa },$$

where *c* is the speed of light. Integrating the electron energy equation by time gives the electron energy:

(A7) $$\epsilon_{e}\left(x,t\right)=C_{0}\times F\left(0,t\right)\times e^{-2x/l_{s}},$$

where $C_{0}=\frac{2A}{n_{e}l_{s}}$ and $F\left(0,t\right)=\int_{0}^{t}I\left(0,\tau \right)d\tau$. The threshold for Ag $\epsilon_{e}\left(0\right)=\epsilon_{b}+w_{e}=7.5$ eV at wavelength λ=1μm, has fluence $F_{th}=0.4$ J/cm^2^.

## Appendix E. Electrostatic Ablation of Ag

Ablation depth, number of ablated atoms, and cooling time for the skin layer for an Ag target. For $F=7.39\times F_{th}=2.96$ J/cm^2^ at laser pulse energy ∼3μJ, $\epsilon_{e}\left(0\right)=55.8$ eV, $v_{e}=4.43\times 10^{8}$ cm/s, the ablation depth $x_{abl}=\frac{l_{s}}{2}\ln\left[\frac{\epsilon_{e}}{\epsilon_{b}+w_{e}}\right]$ is equal to $l_{s}$. The ablation volume, assuming $S_{foc}=10^{-6}$ cm^2^, is $V_{abl}=4.3\times 10^{-12}$ cm^3^, $N_{abl}=2.5\times 10^{11}$ ions $\nu_{ei}^{mom}\approx 0.3\omega_{ei}∼3\times 10^{15}$ s^−1^. The heat diffusion coefficient for Ag $D_{heat}=v_{e}^{2/3}\nu_{ei}^{mom}=21.8$ cm^2^/s and the cooling time $t_{cool}=l_{s}^{2}/D_{heat}=0.85$ ps. The ion removal from these layers occurs under the electrostatic force of hot electrons acting as a gradient of the electronic energy.

Electrostatic ablated Ag ions’ acceleration. $\frac{\partial v_{i}}{\partial t}=\frac{2\epsilon_{e}}{M_{i}l_{s}}=2.3\times 10^{17}$ cm/s^2^ (for $\epsilon_{e}=55.8$ eV, $l_{s}=43$ nm, $M_{i}=107.86$). The average electrons’ velocity is around $10^{8}$ cm/s and the motion of electrons is chaotic after a few electron–electron collisions. The ion’ motion is directional (along the electron pressure gradient when the exciting beam is at the normal to the target surface). Thus, for the Gauss intensity distribution across the focal spot, the main current is directed at the normal at an ion velocity of more than two orders of magnitude lower than that of the electrons.

## Appendix F. Radiation from the Ablated Plasma

The unit vector along the current is r and the direction to the detector located at the distance $R_{0}$ is n. The size of the emitter, *r*, is much smaller than the distance to observation, $r\ll R_{0}$. The emitted wave arrives at the detector as a plane wave. The vector potential of the emitted field reads:

(A8) $$A=\frac{1}{cR_{0}}\int j\times dV.$$

The polarisation of the emitted field depends on the mutual directions of the current and the direction to the observation point:

(A9) $$H=\left(\frac{dA}{dt}\times n\right)/c,\;\;\;E=\left[\left(\frac{dA}{dt}\times n\right)\times n\right]/c,$$

where × is the sign of the vector product. The power of the emitted radiation at the observation point located at the distance $R_{0}$ into the unit of the solid angle Ω is $dI=\left(\frac{cE^{2}}{4\pi }\right)R_{0}^{2}d\Omega$. The distance from the source to the measurement point is larger than the wavelength of emitted radiation and much larger than the size of the source.

**Radiation of the ablated ion current**. The estimate of the vector potential for the ablation case is:

(A10) $$A\approx \frac{1}{cR_{0}}eZN_{abl}v_{i},$$

where $N_{abl}=\int n_{i}dV$ is the full number of ablated ions, $V_{abl}=l_{abl}\cdot S_{foc}$ is the total ablated volume, $N_{abl}=2.5\times 10^{11}$ ions of an ionisation number of an atom Z=1 (for the conditions introduced above in Appendix E for Ag). The estimate for the electric field is:

(A11) $$E\approx \frac{1}{cR_{0}}eZN_{abl}\frac{dv_{i}}{dt}.$$

where the ions’ acceleration is $\frac{\partial v_{i}}{\partial t}=\frac{2\epsilon_{e}}{M_{i}l_{s}}=1.45\times 10^{17}$ cm/s^2^. Taking $R_{0}=1$ cm as an estimate of the distance to the observation point, one gets the field E=580 V/m ($1.93\times 10^{-2}$ CGSE).

## Appendix G. Absence of the Debye Screening

During an interaction time shorter than the ion period (StL) the space and time dependence of the electron temperature is strong, while the electron density remains practically unperturbed, $en_{e}E\approx -n_{e}\nabla T_{e}$. Therefore, assumptions of the static limit and space independence of the electron temperature (isothermal equation of state [7]; see Kruer, p. 14 [7]), essential for deriving the Debye screening, are both inapplicable, $en_{e}E\neq -T_{e}\nabla n_{e}$. The absence of screening is due to the lack of collective interactions of multiple charges.

## Appendix H. Nanomachining: Removal of a Few Mono-Atomic Layers

To remove a few atomic layers with a thickness equal to the mean free path of electrons, the energy of electrons should be slightly above the work function ($l_{mfp}\approx$ nanometre); the laser shall deliver to the Ag surface the energy density (see Appendix D): $F=\frac{w_{e}}{C_{0}}\times \exp\left[\frac{2l_{mfp}}{l_{s}}\right]$ J/cm^2^. For Ag hit by 1000 nm light, only 5% of the surface energy density of 0.247 J/cm^2^ is spent for a few atomic layers’ removal. The number of the atomic layers removed by explosion, i.e., the electron’s mean free path, is controlled by the absorbed surface energy density delivered by the laser. Indeed, the mean free path of electrons in Coulomb collisions is $l_{mfp}\propto \epsilon_{e}^{2}$, while $\epsilon_{e}\propto F$.

The volume and speed of ablation can potentially be improved using StL pulses. They are by an order of magnitude shorter than those typically used in laser micro-machining including the burst ablation mode [23]. The latter achieved the benchmark of a ∼3 mm^3^/min ablation rate using burst-controlled heating and evaporation. Such machining rates are comparable with those in established mechanical tooling equipment. The StL pulses cause energetic ion explosion from the laser-affected surface. Ablation at a high repetition rate (bursts) of StL pulses harnesses the Coulomb explosion of the surface occurring at higher speeds and can potentially improve the throughput of material processing.
